# Dysfunction of NaV1.4, a skeletal muscle voltage-gated sodium channel, in sudden infant death syndrome: a case-control study

**DOI:** 10.1016/S0140-6736(18)30021-7

**Published:** 2018-04-14

**Authors:** Roope Männikkö, Leonie Wong, David J Tester, Michael G Thor, Richa Sud, Dimitri M Kullmann, Mary G Sweeney, Costin Leu, Sanjay M Sisodiya, David R FitzPatrick, Margaret J Evans, Iona J M Jeffrey, Jacob Tfelt-Hansen, Marta C Cohen, Peter J Fleming, Amie Jaye, Michael A Simpson, Michael J Ackerman, Michael G Hanna, Elijah R Behr, Emma Matthews

**Affiliations:** a MRC Centre for Neuromuscular Diseases, UCL Institute of Neurology and National Hospital for Neurology and Neurosurgery, University College London, London, UK; b Cardiology Clinical Academic Group, St George's University of London and St George's University Hospitals NHS Foundation Trust, London, UK; c Division of Heart Rhythm Services, Department of Cardiovascular Medicine, Mayo Clinic, Rochester, MN, USA; d Division of Pediatric Cardiology, Department of Pediatric and Adolescent Medicine, Mayo Clinic, Rochester, MN, USA; e Windland Smith Rice Sudden Death Genomics Laboratory, Department of Molecular Pharmacology & Experimental Therapeutics, Mayo Clinic, Rochester, MN, USA; f Neurogenetics Unit, Institute of Neurology, University College London, London, UK; g Department of Clinical and Experimental Epilepsy, Institute of Neurology, University College London, London, UK; h Chalfont Centre for Epilepsy, Chalfont St Peter, UK; i MRC Human Genetics Unit, MRC Institute of Genetics & Molecular Medicine, University of Edinburgh, Western General Hospital, Edinburgh, UK; j Department of Pathology, Royal Infirmary of Edinburgh, Edinburgh, UK; k Department of Pathology, St George's University Hospitals NHS Foundation Trust, London, UK; l Department of Cardiology, The Heart Centre, Copenhagen University Hospital, Rigshospitalet, Copenhagen, Denmark; m Department of Forensic Medicine, Faculty of Medical Sciences, University of Copenhagen, Copenhagen, Denmark; n Department of Medicine and Surgery, University of Copenhagen, Copenhagen, Denmark; o Department of Histopathology, Sheffield Children's NHS Foundation Trust, Sheffield, UK; p School of Social and Community Medicine, University of Bristol, Bristol, UK; q Department of Medical and Molecular Genetics, Faculty of Life Science and Medicine, King's College London, London, UK

## Abstract

**Background:**

Sudden infant death syndrome (SIDS) is the leading cause of post-neonatal infant death in high-income countries. Central respiratory system dysfunction seems to contribute to these deaths. Excitation that drives contraction of skeletal respiratory muscles is controlled by the sodium channel NaV1.4, which is encoded by the gene *SCN4A*. Variants in NaV1.4 that directly alter skeletal muscle excitability can cause myotonia, periodic paralysis, congenital myopathy, and myasthenic syndrome. *SCN4A* variants have also been found in infants with life-threatening apnoea and laryngospasm. We therefore hypothesised that rare, functionally disruptive *SCN4A* variants might be over-represented in infants who died from SIDS.

**Methods:**

We did a case-control study, including two consecutive cohorts that included 278 SIDS cases of European ancestry and 729 ethnically matched controls without a history of cardiovascular, respiratory, or neurological disease. We compared the frequency of rare variants in *SCN4A* between groups (minor allele frequency <0·00005 in the Exome Aggregation Consortium). We assessed biophysical characterisation of the variant channels using a heterologous expression system.

**Findings:**

Four (1·4%) of the 278 infants in the SIDS cohort had a rare functionally disruptive *SCN4A* variant compared with none (0%) of 729 ethnically matched controls (p=0·0057).

**Interpretation:**

Rare *SCN4A* variants that directly alter NaV1.4 function occur in infants who had died from SIDS. These variants are predicted to significantly alter muscle membrane excitability and compromise respiratory and laryngeal function. These findings indicate that dysfunction of muscle sodium channels is a potentially modifiable risk factor in a subset of infant sudden deaths.

**Funding:**

UK Medical Research Council, the Wellcome Trust, National Institute for Health Research, the British Heart Foundation, Biotronik, Cardiac Risk in the Young, Higher Education Funding Council for England, Dravet Syndrome UK, the Epilepsy Society, the Eunice Kennedy Shriver National Institute of Child Health & Human Development of the National Institutes of Health, and the Mayo Clinic Windland Smith Rice Comprehensive Sudden Cardiac Death Program.

## Introduction

Sudden infant death syndrome (SIDS) is the unexpected death of a seemingly healthy infant. It is the leading cause of post-neonatal infant death in high-income countries^1^ and accounts for 2400 deaths per year in the USA alone.^2^ Incidence varies internationally from approximately 0·1 per 1000 livebirths in Japan and the Netherlands to 0·8 per 1000 in New Zealand.^1^ Death commonly occurs at 2–4 months of age.^2^ Although the cause of death is unknown, several intrinsic and extrinsic risk factors have been identified, including prematurity, male sex, prone sleeping position, and bed sharing.^1^, ^3^ A failure to rouse and respond appropriately to a life-threatening hypoxic event is considered to be a common final pathway.^3^, ^4^, ^5^

Skeletal muscle channelopathies are inherited neuromuscular disorders caused by variants in ion channel genes with an estimated prevalence around one in 100 000.^6^ NaV1.4 is a skeletal muscle voltage-gated sodium channel, encoded by the gene *SCN4A*, that is crucial for the generation of action potentials and excitation of muscle. Gain-of-function variants in NaV1.4 typically cause myotonia or periodic paralysis.^7^ An infantile myotonic phenotype characterised by severe respiratory compromise has been linked to such gain-of-function variants. Affected infants have brief, recurrent episodes of life-threatening respiratory muscle myotonia causing apnoea, hypoxia, and cyanosis.^8^, ^9^, ^10^, ^11^ Intensive care and tracheostomy are often required.^9^ The onset of apnoea can be delayed by up to 10 months after birth, when infants are seemingly healthy (appendix p 3). Two deaths from respiratory complications have been reported.^8^, ^9^

Loss-of-function variants in NaV1.4 have been reported in patients with congenital myasthenic syndrome and congenital myopathies.^12^, ^13^, ^14^ These patients had evidence of respiratory compromise including sudden brief attacks of apnoea that required ventilator support.^12^

**Research in context**

**Evidence before this study**

Sudden infant death syndrome (SIDS) is the sudden and unexpected death of an apparently healthy infant. It is the leading cause of post-neonatal infant death in high-income countries. Placing infants to sleep in a prone position is associated with a higher risk of sudden death but unidentified risk factors remain. We searched PubMed for papers published in English up to March 1, 2017, reviewing the cause or risk of SIDS using the terms “sudden infant death”, “SIDS”, “sudden unexpected death in infancy”, and “risk factors”. Multiple risk factors have been proposed on the basis of epidemiological, pathological, and genetic studies that include the notion of a vulnerable infantile period due to immature homoeostatic and autonomic regulatory pathways or genetic variation—eg, cardiac channel gene variants. Respiratory failure is ultimately believed to contribute to death.

**Added value of this study**

To our knowledge, this study is the first to consider direct failure of the respiratory muscles due to dysfunction of the skeletal muscle ion channel NaV1.4 in the pathogenesis of SIDS. We compared rare variants in the *SCN4A* gene, which codes for the NaV1.4 channel, among infants who had died of SIDS with variants from ethnically matched controls. We also studied the functional consequences of rare variants in both cases and controls using a heterologous cell system. Only infants who had died from SIDS carried variants that significantly disrupted channel function.

**Implications of all the available evidence**

Our data are compatible with the clinical features of SIDS and provides new mechanistic clues to death. Developmental regulation of the sodium channel and respiratory muscle fibre types might affect the risk of SIDS. Future research is required to define this relationship, and our findings should be retested in similar and other ethnic groups. Sodium channel dysfunction found in muscle channelopathies can be treated. Studies should assess whether such treatments could ameliorate the risk of sudden death for infants carrying *SCN4A* gene variants.

These fatal and life-threatening *SCN4A* respiratory phenotypes are compatible with the model of SIDS pathogenesis. We hypothesised that rare functionally deleterious *SCN4A* variants might be over-represented in infants who had died of SIDS.

## Methods

### Study design and participants

We did a case-control study, comparing infants who had died from SIDS with ethnically matched adult controls. We first utilised a small cohort of UK white European SIDS cases and then replicated our findings in a larger cohort of USA samples. We obtained fully anonymised exome data from all SIDS cases from an international exome sequencing collaboration. The cohort consisted of coroners' cases from the UK and cases referred to coroners, medical examiners, or forensic pathologists from six ethnically and geographically diverse populations in the USA. We defined SIDS as the sudden death of a seemingly healthy infant that remained unexplained despite a thorough investigation of the scene and circumstances of death, a comprehensive post-mortem examination including microbiology and histopathology performed by a pathologist or medical examiner with paediatric training, and a multiprofessional review of the available information.^15^, ^16^ The control cohort comprised exome sequencing data from adults of European ancestry without a history of cardiac, neurological, or respiratory phenotypes reported to the same international collaboration as the cases, with no further inclusion or exclusion criteria.

Genetic studies were undertaken on anonymised samples as approved by the National Research Ethics Service—Wandsworth (reference: 10-H0803-73) and the Mayo Clinic Institutional Review Board.

### Procedures

Whole exome sequencing for both cases and controls was done using 1·5–3 μg of genomic DNA. Samples from the UK were sequenced at King's College London using the Sure Select XT Human All Exon v5 Target Enrichment System (Agilent, Santa Clara, CA); samples from the USA were sequenced at the Mayo Clinic with the Sure Select XT Human All Exon + UTR v5 Target Enrichment System (Agilent, Santa Clara, CA). DNA libraries were prepared according to manufacturer's protocols. 100 base pair paired end sequencing was performed on the Illumina HiSeq 2500 platform.

Sequencing reads from exome sequencing of cases and controls were aligned to the GRCh37 human reference genome using NovoAlign. Variant calling, multi-sample genotyping, and variant quality recalibration was done with GATK. Coverage across the protein-coding regions of the exome and *SCN4A* specifically were assessed using the Bedtools package. A set of 3847 common variants located outside of regions of the genome where there is extensive linkage disequilibrium^17^ were used to estimate relatedness within the study cohort and ethnic ancestry alongside the control group using the King software package.^18^ Cases and controls that clustered closely with individuals from the Caucasian European population from the 1000 Genomes Project were included in the downstream analysis to compare individuals from the same ethnic genetic background.

Variants identified within the *SCN4A* locus (chromosome 17: 62015914–62050278) were annotated with their predicted effect on the *SCN4A* transcript NM_000334.4 (GenBank) and allele frequencies derived from the Exome Aggregation Consortium. The analysis focused on predicted protein-altering alleles that were novel or with an allele frequency less than 0·00005. Variants were confirmed by Sanger sequencing using methods previously described.^19^ We assessed the effect of intronic variants on splicing using Human Splicing Finder.^20^

For mutagenesis and in-vitro transcription, we used the human *SCN4A* expression clone, pRc/CMV-hSkM1,^21^, ^22^ based on accession M81758.1, which we have previously used for functional expression.^14^, ^23^, ^24^ Site-directed mutagenesis was done with the QuikChange kit (Agilent) and confirmed by sequencing the entire insert.

All identified rare *SCN4A* mis-sense variants were characterised by patch clamp heterologous expression studies to evaluate the consequence on NaV1.4 channel activity. Functional properties of mutant NaV1.4 channels in expression systems show excellent correlation with predicted changes in excitability of native cells and the clinical phenotype of the patient carrying the variant.^7^, ^12^, ^13^, ^14^, ^25^ We assessed expression in HEK293 cells, which do not express endogenous sodium channels.^14^, ^26^ The cells were incubated in a 1·9 cm^2^ well with transfection mixture consisting of 0·5 μg of wild type or mutant human *SCN4A* plasmid, 50 ng of plasmid coding for copGFP, 1·5 μl Lipofectamine 2000 (ThermoFisher) in 100 μL of Opti-MEM (ThermoFisher) for 16–24 h. Wild type *SCN4A* plasmid was included in every set of transfections to control for transfection efficacy. Two independent DNA preparations were used for the Glu1520Lys variant to control for the effect of the quality of the preparation on reduced expression levels.

For whole cell patch clamping, HEK293 cells with green fluorescence were voltage clamped at room temperature 48–72 h after transfection using an Axopatch 200B Amplifier (Axon Instruments), NI-6221 (National Instruments), or Digidata 1440B digitiser and pClamp software (Axon Instruments). Holding voltage was −80 mV. The current–voltage and conductance–voltage relationships were fitted with the Boltzmann equation to estimate the midpoint and the slope factor of the voltage dependence:

$$\text{G}\;\text{or}\;\text{I}=\text{A}+\frac{\text{B}-\text{A}}{1+\text{exp}\frac{{\text{V}}_{1/2}-\text{V}}{{\text{V}}_{\text{slope}}}}$$

where G is electrical conductance, I is current, A is the maximum amplitude, B is the minimum amplitude, V_1/2_ (voltage of half-maximal activation or inactivation) is the voltage where amplitude is

$$\frac{\text{A}+\text{B}}{2}$$

and V_slope_ is the slope factor. The time constant of recovery from inactivation (τ_recovery_) and of onset of fast inactivation (τ_inactivation_) were derived by fitting single or double exponential functions, respectively, to the timecourse data. We analysed only the fast component (routinely >95% of the total amplitude) of the double exponential function. The series resistance error was kept below 5 mV.

We compared the number of ultra-rare and functionally deleterious *SCN4A* gene variants between cases and controls using a one-tailed Fisher's Exact test. We used Kruskal–Wallis rank sum test with Dunn's pairwise multiple comparisons or a one-way ANOVA with Games-Howell's post-hoc test to compare the mean of each variant against the wild-type mean. We used a Bonferroni correction to correct for multiple comparisons across all parameters.

We analysed the data using pClamp, Microsoft Excel, Origin, Prism, and SPSS 24 software.

### Role of the funding source

The funder of the study had no role in study design, data collection, data analysis, data interpretation, or writing of the report. The corresponding author had full access to all the data in the study and had final responsibility for the decision to submit for publication

## Results

We obtained exome data from 427 SIDS cases (95 from the UK and 332 from the USA) and 729 controls. After sequence alignment, seven cases were excluded from further analysis because less than 75% of the Gencode-defined protein-coding exome was covered by fewer than 20 reads. One further case from a half-sibling pair was excluded from downstream analysis. 141 cases were not white European and were removed from the final analysis. A total of 278 cases (84 from the UK, 194 from the USA) and 729 controls were included in the downstream analysis.

**Figure 1:**
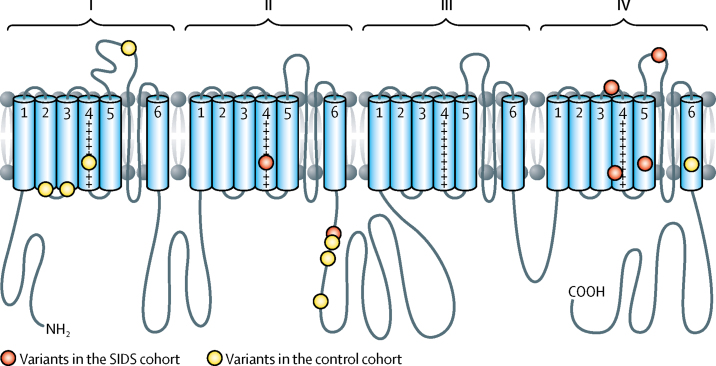
The location of mutations in the NaV1.4 channel The location of mutations in the NaV1.4 channel Transmembrane helices S1–S6 are labelled in domains I–IV. The S4 helices contain positively charged arginine residues (+). The S1–S4 helices form voltage-sensing domains. The S5–S6 helices are pore-forming. The variants in the SIDS cohort (from N-terminus to C-terminus) are: Ser682Trp, Gly859Arg, Val1442Met, Arg1463Ser, Met1493Val, and Glu1520Lys. The variants in the control cohort (from N-terminus to C-terminus) are: Arg179Gln, Arg190Trp, Leu227Phe, Asp334Asn, Gly863Arg, Ala870Thr, Met897Lys, and Val1590Ile. SIDS=sudden infant death syndrome.

More than 81% of the DNA sequences comprising the protein coding regions and associated splice sites of the *SCN4A* gene were covered by at least 20 reads in each individual in the study cohort and more than 90% were covered by at least ten reads (appendix p 5). We identified rare alleles in the *SCN4A* gene in six (2%) of 278 infants of white European descent who died of SIDS and in nine (1%) of 729 ethnically matched controls (p=0·21, one tailed Fisher's Exact test; table 1, figure 1). One of the NaV1.4 variants present in an infant who died from SIDS (Arg1463Ser) was also recorded in the UK national skeletal muscle channelopathy database in an adult patient with myotonia.

**Table 1:** Novel and rare *SCN4A* variants in SIDS cases of European ancestry and ethnically matched controls

	**Exome Aggregation Consortium allele frequency**	**Functional expression results**	**Position in channel**	**Age (months)**	**Sex**	**Co-sleep?**	**Evidence of URTI?**	**Term**	**Sleep position**	**Exposed to cigarette smoke?**
**SIDS cases**										
Ser682Trp (2045C→G)	0·00002626	Gain of function	DII/S4	3	Male	Yes	No	NA	Prone	Yes
Gly859Arg (2575G→A)	0·00001756	Wild type like	DII–III cytoplasmic loop	3	Female	Yes	No	Full	Prone	NA
Val1442Met (4324G→A)	0·00001025	Loss of function	DIV/S3-4 extracellular loop	5	Male	No	No	Premature	Supine	No
Arg1463Ser (4387C→A)	0·00000832	Gain of function	DIV/S4	3	Male	Yes	No	Full	Side	Yes
Met1493Val (4477A→G)	Novel	Wild type like	DIV/S5	3	Female	No	No	NA	Prone	NA
Glu1520Lys (4558G→A)	Novel	Loss of function	DIV/S5–6 pore-forming loop	2	Male	NA	Yes	NA	NA	NA
**Controls**										
Intron change (393-1C→T)	Novel	NA	NA	..	..	..	..	..	..	..
Arg179Gln (G536A)	0·00001862	Wild type like	DI/S2–3 cytoplasmic loop	..	..	..	..	..	..	..
Arg190Trp (C568T)	Novel	Wild type like	DI/S2–3 cytoplasmic loop	..	..	..	..	..	..	..
Leu227Phe (C679T)	0·00004441	Wild type like	DI/S4	..	..	..	..	..	..	..
Asp334Asn (G1000A)	0·00002484	Wild type like	DI/S5–6 extracellular loop	..	..	..	..	..	..	..
Gly863Arg (G2587A)	0·00001581	Wild type like	DII–III cytoplasmic loop	..	..	..	..	..	..	..
Ala870Thr (G2608A)	Novel	Wild type like	DII–III cytoplasmic loop	..	..	..	..	..	..	..
Met897Lys (T2690A)	Novel	Wild type like	DII–III cytoplasmic loop	..	..	..	..	..	..	..
Val1590Ile (G4768A)	0·000008278	Wild type like	DIV/S6	..	..	..	..	..	..	..

Functional expression results refer to the patch clamp data. NA=not available. URTI=upper respiratory tract infection. D=domain. S=segment.

Using our whole exome sequencing data, we also determined whether any of the six SIDS cases with a rare *SCN4A* variant carried a rare variant in any of 90 genes associated with inherited cardiac condition including those associated with SIDS (appendix pp 8–9). Only one such participant did (with variant Ser682Trp in *SCN4A*). In addition to the *SCN4A* variant, this individual had variants in *SCN5A* (Gly333Arg) and in *NEXN* (Met38Thr).

**Figure 2:**
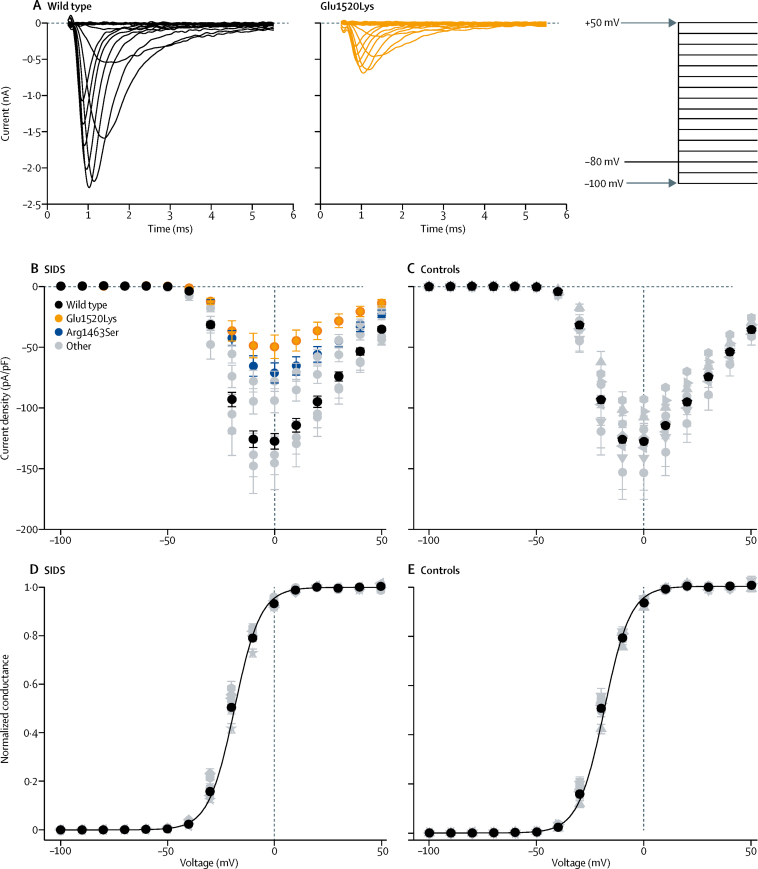
Activation properties of NaV1.4 variants (A) Representative current traces of wild type, and Glu1520Lys channels in response to test voltages in 10 mV increments from −100 mV to + 50 mV (the voltage protocol is shown on the right). Dashed lines indicate 0 current level. (B,C) Peak current density in response to test voltages ranging from −100 to + 50 mV in 10 mV increments is plotted against the test voltage for variants in the SIDS cohort (B) and in controls (C). Current densities of Glu1520Lys (orange) and Arg1463Ser (blue) were significantly lower than wild type (black; appendix p 6–7). Data for the other variants that did not differ significantly from wild type are shown in grey. (D,E) Voltage dependence of activation. Normalised conductance (peak current/[test voltage – reversal voltage]) is plotted against the test voltage for variants in the SIDS cohort (D) and in controls (E). Individual data were normalised to maximum and minimum amplitude of the Boltzmann fit and averaged. The solid lines represent the fit of Boltzmann equation to mean data. Voltage of half-maximal activation (V_1/2_) did not differ significantly from wild type (black) for any of the variants (appendix p 6–7) and the data are illustrated with grey symbols. The statistical analysis is shown in the appendix (p 6).

**Figure 3:**
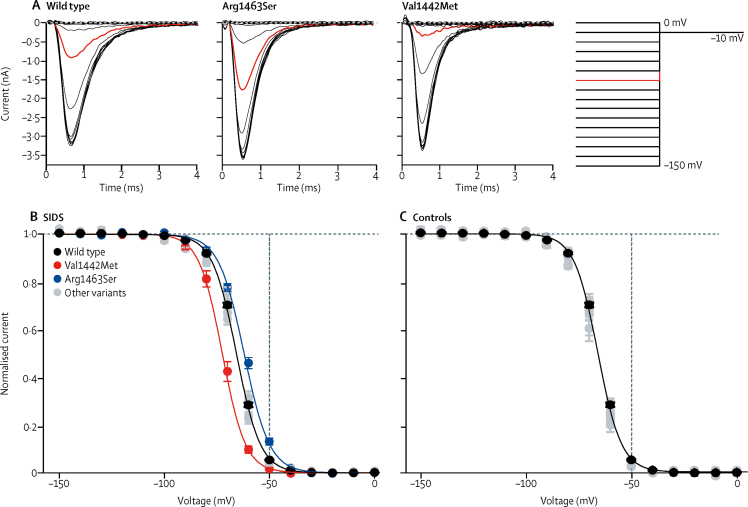

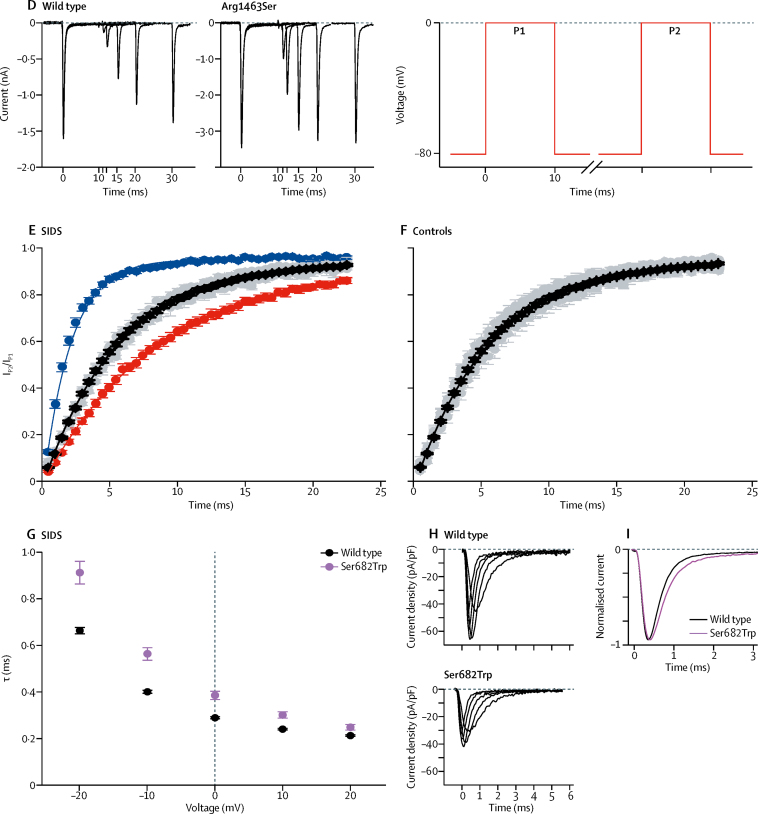
Fast inactivation properties of NaV1.4 variants (A–C) Voltage dependence of fast inactivation. (A) Representative current traces in response to tail voltage step to −10 mV following 150 ms pre-pulse voltage steps ranging from −150 mV to 0 mV in 10 mV increments for wild type, Arg1463Ser, and Val1442Met variants. First 5 ms of the voltage step to −10 mV are shown. Current response following pre-pulse step to −60 mV is highlighted in red. The voltage protocol is shown to the right. Dashed lines indicate 0 current level. (B,C) The peak tail current amplitude at −10 mV is plotted against the pre-pulse voltage for variants in the SIDS cohort (B) and in controls (C). Individual data were normalised to maximum and minimum values of the Boltzmann equation and averaged. Voltage of half-maximal inactivation was shifted significantly to more hyperpolarised voltages for Val1442Met channels (red) and to more depolarised voltages for Arg1463Ser channels (blue). The solid lines represent the fit of Boltzmann equation to mean data for wild type, Val1442Met and Arg1463Ser channels. The data for the variants that did not differ from wild type channels are shown in grey. (D–F) Recovery rate from fast inactivation. (D) Representative current traces for wild type and Arg1463Ser channels illustrating the recovery from inactivation. Channels were inactivated by a 10 ms voltage step to 0 mV and then stepped to recovery voltage of −80 mV for an increasing duration of time. A second voltage step to 0 mV was then applied to see how much the channels had recovered from fast inactivation. Only traces with 0, 1, 2, 5, 10, and 20 ms duration at recovery voltage of −80 mV are shown. The voltage protocol is shown to the right. (E,F) The peak current amplitude during the second step (P2) is divided by the peak current amplitude during the first voltage step (P1) and plotted against the duration of the recovery period at −80 mV. Data for variants in the SIDS cohort (E) and in controls (F) is colour coded as in (B) and (C). The solid lines represent fit of exponential function to the mean data. (G–I) Rate of fast inactivation. (G) Time constant (τ) of inactivation at voltages ranging from −20 mV to +20 mV for wild type (black) and Ser682Trp (magenta) channels. The voltage protocol was as in figure 2A. Only the time constant of the fast component that carries roughly 95% of the amplitude of the inactivating current is analysed. (H) Representative current traces at −20 mV to +20 mV for wild type (top) and Ser682Trp (bottom) channels, showing the slower inactivation of the Ser682Trp variant. (I) Overlay of mean normalised current traces for wild type (black) and Ser682Trp (magenta) channels in response to voltage step to 0 mV.

Heterologous expression studies showed that four of the six variants in infants who died of SIDS, but none of the coding variants in controls, disrupted *SCN4A* function. The voltage dependence of activation for each of the identified alleles was not significantly different from the wild type allele except for a small increase in slope factor (V_Slope_) for variant Ser682Trp (figure 2D, 2E, table 2). However, the current density in response to a voltage step to 0 mV was significantly reduced for two of the six variants identified in the case cohort (Glu1520Lys, Arg1463Ser; figure 2A, 2B) and none in controls (figure 2C). In addition, the Arg1463Ser variant showed a shift in the voltage of half-maximal fast inactivation to + 3·6 mV (figure 3A, 3B, table 2) and the recovery from inactivation was almost three-times faster than in the wild type channel (figure 3D, 3E, table 2). Furthermore, fast inactivation of Val1442Met was enhanced as the voltage of half-maximal inactivation was 6·6 mV lower in the variant than in wild type and recovery from inactivation was 1·5 times slower (figure 3A, 3B, table 2). The time course of open state inactivation was significantly slower for the Ser682Trp variant at 0 mV (figure 3G–I, table 2). Voltage dependence of inactivation (figure 3C), time constant of recovery from inactivation (figure 3F), and the time constant of inactivation (table 2) of the variants in control cohort did not differ significantly from wild type. The intron variant in the control cohort was predicted to have no effect on splicing (Human Splicing Finder).

**Table 2:** Biophysical parameters of NaV1.4 variants

		**Activation**					**Fast inactivation**				
		N	I_Peak_ at 0 mV (pA/pF)	N	V_1/2_(mV)	V_slope_(mV)	V_1/2_(mV)	V_slope_(mV)	τ_Inactivation_ at 0 mV (ms)	N	T_Recovery_(ms)
Wild type		149	−127·5 (6·4)	146	−19·5 (0·2)	6·4 (0·1)	−65·3 (0·3)	5·4 (0·0)	0·30 (0·00)	105	5·63 (0·14)
SIDS cohort											
	Ser682Trp	19	−94·2 (9·9)	17	−21·2 (0·7)	7·2 (0·2)*	−67·0 (0·6)	5·9 (0·2)	0·40 (0·02)*	17	6·15 (0·33)
	Gly859Arg	18	−138·5 (16·4)	17	−20·2 (0·8)	5·7 (0·2)	−64·2 (0·6)	5·1 (0·1)	0·31 (0·01)	17	5·20 (0·30)
	Val1442Met	14	−145·5 (21·7)	14	−21·7 (0·8)	6·3 (0·2)	−71·9 (1·0)*	5·2 (0·1)	0·27 (0·01)	13	8·51 (0·46)*
	Arg1463Ser	27	−71·3 (8·3)*	25	−17·4 (0·5)	6·7 (0·2)	−61·7 (0·6)*	6·3 (0·1)*	0·31 (0·01)	19	1·95 (0·09)*
	Met1493Val	17	−78·2 (8·3)	17	−19·2 (0·5)	6·7 (0·3)	−65·3 (0·8)	5·5 (0·2)	0·29 (0·01)	7	5·76 (0·47)
	Glu1520Lys	39	−49·8 (9·7)*	28	−20·3 (0·4)	6·3 (0·1)	−66·3 (0·5)	5·4 (0·2)	0·33 (0·01)	20	5·63 (0·29)
Control cohort											
	Arg179Gln	11	−117·6 (11·0)	11	−19·3 (0·6)	6·5 (0·2)	−65·1 (0·7)	5·1 (0·1)	0·25 (0·01)	11	4·73 (0·30)
	Arg190Trp	14	−153·4 (21·8)	14	−20·7 (0·5)	6·4 (0·2)	−64·9 (0·5)	5·3 (0·1)	0·29 (0·01)	13	5·09 (0·20)
	Leu227Phe	12	−107·7 (16·1)	12	−17·6 (0·4)	6·5 (0·2)	−67·8 (0·8)	5·6 (0·1)	0·33 (0·02)	10	5·33 (0·30)
	Asp334Asn	10	−141·0 (26·6)	10	−21·0 (0·9)	6·4 (0·2)	−66·4 (0·8)	5·3 (0·1)	0·27 (0·01)	9	5·07 (0·30)
	Gly863Arg	12	−124·6 (15·8)	12	−20·3 (0·8)	6·3 (0·1)	−66·1 (0·7)	5·2 (0·2)	0·29 (0·01)	10	5·71 (0·21)
	Ala870Thr	16	−132·6 (20·0)	15	−20·0 (0·6)	6·4 (0·1)	−65·7 (0·6)	5·0 (0·1)	0·29 (0·01)	14	6·29 (0·55)
	Met897Lys	10	−102·8 (23·3)	9	−20·4 (0·7)	6·8 (0·3)	−67·7 (1·3)	5·5 (0·2)	0·31 (0·02)	7	6·21 (0·54)
	Val1590Ile	8	−92·8 (13·2)	8	−20·7 (0·6)	6·8 (0·2)	−65·8 (0·9)	5·4 (0·2)	0·30 (0·02)	7	5·73 (0·61)

Data are mean (SE). N indicates the number of cells recorded from. I_Peak_ includes all cells with current amplitude larger than 0·1 nA. Only cells with I_Peak_larger than 0·5 nA were included in the analysis of the other biophysical properties. Voltage dependence of activation and fast inactivation, and time constant of open state fast inactivation were all analysed in the same recording for each cell.

* p value compared with wild type is less than the Bonferroni threshold (p=0·00051; 98 tests from 14 variants and seven parameters). The appendix shows uncorrected p values (p 6–7). I_peak_=peak current density. V_1/2_=the voltage of half-maximal activation or inactivation. V_slope_=slope factor. τ_Inactivation_=time constant of open state fast inactivation. T_Recovery_=time constant of recovery from inactivation.

We initially tested our hypothesis in a UK cohort. Genetic data were available from 84 white Europeans. Of these UK cases, one carried a functional *SCN4A* variant, Arg1463Ser. This was an underpowered cohort but this positive finding warranted testing our hypothesis in a larger cohort. We then analysed genetic data in a second cohort that included 194 DNA samples from cases of white European descent from the USA to assess whether our finding could be replicated. Three (1·5%) of 194 infants in this second cohort carried functional *SCN4A* variants, compared with none of 729 ethnically matched controls (p=0·009 Fisher's exact test).

Taking the UK and US cohorts of 278 cases together, four of the six rare variants in the SIDS cohort had significant differences in channel gating compared with wild type *SCN4A* (Figure 2, Figure 3, and table 2); by contrast, none of the variants in controls had any differences from wild type *SCN4A* (p= 0·011, Fisher's exact test). Therefore, four of 278 SIDS cases had a functionally disruptive *SCN4A* missense variant (1·4%) compared with none of the 729 ethnically matched controls (p=0·0057, Fisher's exact test). Even if the intron variant in the control cohort is assumed to be deleterious, functional variants remain over-represented in the SIDS group (p=0·02).

## Discussion

We identified rare *SCN4A* variants in both Caucasian European infants who died of SIDS and in living adult controls. We showed that rare variants that alter NaV1.4 channel function in a similar fashion to known pathogenic *SCN4A* variants were over-represented in infants who had died of SIDS compared with ethnically matched controls.

Alterations in sodium channel function in SIDS are qualitatively similar to those reported in infants with life-threatening respiratory events.^9^, ^12^, ^14^ Both gain-of-function and loss-of-function *SCN4A* variants can cause severe respiratory presentations in infancy and some of these cases have had a fatal outcome.^8^, ^9^, ^14^ In our study, only the *SCN4A* variants present in white SIDS cases caused significant alterations in channel function compared with wild type. By contrast, the variants we discovered in control adults did not alter channel function. The Arg1463Ser and Ser682Trp variants caused impaired fast inactivation that resulted in a predominantly gain-of-function effect in the NaV1.4 sodium channel. Similar gain-of-function alterations in channel function are the hallmark of *SCN4A* variants in patients with myotonia, including in infants with severe respiratory complications.^25^ The pathogenic role of Arg1463Ser is also supported by its presence in a patient under our care (EM, MGH) with generalised myotonia. The heterozygous loss-of-function variants we discovered in the SIDS cohort caused either substantial enhancement of fast inactivation (Val1442Met) or reduced current density (Glu1520Lys). Compound heterozygous or homozygous loss-of-function NaV1.4 variants (including the previously reported Val1442Met variant) cause myasthenic syndrome and congenital myopathy.^12^, ^14^ Some of these patients need respiratory support.^12^, ^14^ These observations support the notion that disrupted sodium channel function in the respiratory muscles can contribute to life-threatening events.

Developmental regulation of *SCN4A* expression and muscle fibre typing might define a period of SIDS vulnerability. The SIDS infants in our cohort would have appeared healthy and asymptomatic before death. We propose that the functionally deleterious variants we have identified can explain this apparent healthiness of infants and also represent a major risk factor for SIDS. The gain-of-function change we observed in the infant with Arg1463Ser is consistent with previously reported *SCN4A* variants (appendix pp 1–4) that, after a period of apparent clinical normality, cause myotonia of the laryngeal and respiratory muscles precipitating an abrupt onset apnoeic crisis. For those with heterozygous loss-of-function variants (Val1442Met and Glu1520Lys) and the milder gain-of-function variant (Ser682Trp), the level of channel perturbation shown in our study might not be sufficient to cause respiratory failure but it could be a significant contributing factor. Baseline respiratory function could be normal but the presence of these variants might impair the ability of respiratory muscles to mount and maintain rapid and forceful contraction in response to hypoxia.^4^

Two developmental factors might combine with the functional effects of the *SCN4A* variants we identified to render respiratory muscles more susceptible to contractile failure—eg, in response to hypoxia. First, developmental alterations in skeletal muscle sodium channel expression: during embryogenesis two different sodium channel isoforms, the cardiac isoform NaV1.5 (encoded by *SCN5A*) and the skeletal muscle isoform NaV1.4 are expressed in skeletal muscle although NaV1.4 predominates. The expression of NaV1.5 progressively decreases over the first 2 years after birth. The expression of NaV1.4 progressively increases after birth but the level before the age of 5 years is 25–40% of that seen in adulthood.^27^ The presence of NaV1.5 expression could compensate to some degree for variant-induced NaV1.4 dysfunction and to the delay in onset of symptoms reported in patients with myotonia who have NaV1.4 dysfunction.^27^

Low expression of NaV1.4 in infantile muscle is likely to be particularly crucial in fast twitch respiratory muscles^28^, ^29^, ^30^ because their ability to maintain the amplitude of successive action potentials when under increased demand—eg, in the presence of hypoxia—is dependent on the density of sodium channels.^31^, ^32^ This suggestion is supported by the observation that the capacity of muscle fibres from NaV1.4 heterozygous null mice to generate sustained action potentials diminishes with repeated stimulation.^33^ The enhanced sodium channel inactivation we observed with the Val1442Met variant will reduce channel availability, which could be particularly detrimental during such high frequency stimulation.^13^

Second, developmental alterations in respiratory muscle fibre typing: the proportion of fast and slow twitch fibres in respiratory muscles is regulated developmentally. Fast twitch fibres that rely more heavily on the density of sodium channels predominate in these muscles in infants, compared with those older than 2 years of age.^34^

These developmental changes in sodium channel expression patterns and in muscle fibre type composition could define a period of vulnerability in early life when the respiratory and laryngeal muscles are particularly dependent on NaV1.4 channel function. Our data support the notion that the NaV1.4 dysfunction caused by the *SCN4A* variants will exacerbate this vulnerability.

Several related concurrent events are thought necessary for sudden infant death.^35^ A triple risk hypothesis^36^, ^37^ states that for death to occur there must be a convergence of factors: a vulnerable infant, a critical period of development, and an external stressor. Our data suggest that the presence of an *SCN4A* gene variant that impairs sodium channel function exacerbates an infant's vulnerability. In addition, the notion of developmental regulation of respiratory muscle fibre types and muscle sodium channel expression may define a critical period of development when they are particularly vulnerable to respiratory stressors. Fasting-induced alterations in extracellular potassium that might occur during sleep could further exacerbate the deleterious gain-of-channel function effects. Our data are consistent with the notion that risk factors combine to increase the probability of SIDS occurring, but may not be the sole cause of death. This hypothesis concurs with the presence of some very rare variants with effects on NaV1.4 function in the Exome Aggregation Consortium database. If the *SCN4A* gene changes with an effect on channel function were individually or universally fatal, they would be unlikely to be present in living controls. Parallels can be made with studies of variants in *SCN5A* cardiac sodium channel and the β subunits of Nav1.5 in infants who die from SIDS.^38^, ^39^ Functional variants in these genes can predispose patients to cardiac arrhythmia but they are also rarely found in the Exome Aggregation Consortium database, indicating that they contribute to the probability of death occurring but might not define it.^40^ One of our SIDS cases (Ser682Trp) did also carry ultra rare variants in the *SCN5A* (Gly333Arg) and *NEXN* (Met38Thr) cardiac genes. Although other variants in *SCN5A* have been linked to arrhythmia syndromes and SIDS, this specific variant has not been reported in either of these presentations and has not been functionally characterised. It occurs in an extracellular loop of the protein that is not a critical domain for protein function but the aminoacid is conserved across species.^41^ Variants in *NEXN* have been associated with inherited cardiomyopathy^42^ but no link with the risk of SIDS has been established. The significance of these variants is therefore unknown. Our findings are the first direct evidence that a primary defect in skeletal muscle excitability might be a risk factor for SIDS.

Our study had several limitations. It was restricted to the examination of *SCN4A* variants in European white individuals. SIDS occurs in all ethnicities at varying rates and our study should be replicated in other ethnic groups. Because of the anonymity of our samples, little clinical data were available and other family members could not be tested. Our functional analyses were also restricted to effects on sodium channel gating properties in vitro.

Our data show that functionally disruptive *SCN4A* variants are over-represented in SIDS. We propose that such variants impair the ability of respiratory muscles to respond to hypoxia. The developmental regulation of sodium channel expression and skeletal muscle fibre type composition may be factors in the period of susceptibility. Although we have studied two cohorts, which together form one of the largest SIDS cohorts reported,^43^ replication in other cohorts is needed to evaluate the potential role of *SCN4A* variants as a risk factor in SIDS. In addition, prospective studies with direct family involvement will help to define the clinical correlation of in-vitro muscle sodium channel dysfunction with SIDS.

Sodium channel blockers can reduce the frequency and severity of myotonia in patients with gain-of-function NaV1.4 variants,^44^ including those with severe infantile myotonia who have life-threatening respiratory compromise (appendix pp 1–4).^8^, ^9^ Furthermore, acetazolamide, which may be effective in loss-of-function *SCN4A* channelopathies,^12^, ^45^ has been reported to abolish attacks of respiratory and bulbar weakness in a patient with myasthenic syndrome associated with the Val1442Glu loss-of-function variant.^12^ These data suggest that drug treatment could reduce the risk of *SCN4A* variants in siblings of an index case, but this requires further detailed evaluation. In conclusion, our data suggest that *SCN4A* variants are a genetically and mechanistically plausible risk factor for SIDS.

For the **Exome Aggregation Consortium** see http://exac.broadinstitute.org/

For the **Human Splicing Finder** see http://www.umd.be/HSF3/HSF.shtml
