# Supplementary material for: Dysfunction of NaV1.4, a skeletal muscle voltage-gated sodium channel, in sudden infant death syndrome: a case-control study
Source: Lancet. 2018 Apr 14;391(10129):1483–92. doi: 10.1016/S0140-6736(18)30021-7 (PMC5899997; doi:10.1016/S0140-6736(18)30021-7)
Supplement: Supplementary appendix [file mmc1.pdf]

THE LANCET

Supplementary appendix

This appendix formed part of the original submission and has been peer reviewed. We post it as supplied by the authors.

Supplement to: Männikkö R, Wong L, Tester D J, et al. Dysfunction of NaV1.4, a skeletal muscle voltage-gated sodium channel, in sudden infant death syndrome: a case-control study. *Lancet* 2018; published online March 28. http://dx.doi.org/10.1016/S0140-6736(18)30021-7.

	Clinical features	Sex	Mutation	Refs.
1	APGAR scores 10/10. A few hours later, tachypnea and abdominal distension, admitted ICU. After recovery, apnea and hypoxic episodes recurred 48 h later.<br>Dysmorphic features: high forehead, down slanting palpebral fissures, low-set ears, short neck, and high arched palate. Congenital hip dislocation.<br>ECG/echo normal. Potassium normal. CK 142.<br>Recurrent apnea from 1 to 10 times per day. Typical prodrome included profuse sweating. Arm stiffness and loss of consciousness present during all attacks, followed by severe desaturation and cyanosis. Ended with general muscle weakness and heavy inspiration which provided a rapid return to normal vital signs.<br>Failure to thrive despite PEG. Severe GORD and constipation. Psychomotor delay. Truncal hypotonia with peripheral hypertonia and muscle hypertrophy. Reflexes present.<br>EMG profuse myotonia. Legs in cold water led to immediate attack of fainting and weakness.<br>Muscle biopsy non-specific. No vacuoles. Some excess mitochondria.<br>No response to CBZ. Relatively good response to mexiletine for several months but ultimate recurrence of symptoms. Death from respiratory arrest in conjunction with LRTI.	F	SCN4A<br>N1297K<br>De novo	(1)
2	Episodic stridor when bottle fed in first days of life.		SCN4A<br>G1306E<br>De novo<br>+ CLCN1<br>M485V	(2)
3	APGAR score 10. Intermittent stridor since birth. ICU admission at 16 days with laryngospasm and life threatening apnea.<br>Daily attacks of laryngospasm associated with generalized stiffness, cyanosis, and bradycardia<br>Examination normal between attacks apart from occasional generalized stiffness and stridor.<br>Tracheostomy at 3 months.<br>6 months muscle hypertrophy (mini athlete), clinical and EMG myotonia. Genetic dx made and carbamazepine started. 14months tracheostomy removed. 18months normal development and growth.	M	SCN4A<br>G1306E<br>De novo	(3)
4	APGAR score 10. Intermittent stridor since birth. ICU at 3days, daily apneas with ALTEs<br>At 2 months, slow initiation of bottle-feeding and intermittent stridor.<br>Apneic episodes associated with initial stridor + transient stiffness of upper limbs, followed by bradycardia, pallor, and hypotonia, suggestive of obstructive apneas, confirmed by polysomnography.<br>Spontaneous improvement in apneas at 3months.<br>At 7 months, expressionless face, unable to sit, and displayed slow limb movements.<br>Then muscular appearance and cold exacerbated myotonia more obvious.<br>Mexiletine at 11 months – laryngospasms stopped within 1 week, polysomnography normalized, motor abilities improved, allowing independent walking at 16 months.	M	SCN4A<br>G1306E<br>De novo	(3)
5	APGAR score 4/9/9/.Immediate respiratory distress at birth, intubation and transfer to ICU. Spontaneous ventilation possible after 3 days.<br>O/E generalized hypertonia, including facial and eyelid muscles. Episodic stridor noted. Spontaneous or reflex movements’ poor, with	M	SCN4A<br>Ala799Ser<br>De novo	(3)

	sucking and swallowing automatisms, requiring NG tube feeding.<br>Brief episodes of severe apnea occurred several times a day, resulting in cyanosis and transient hypoxia. Death from respiratory arrest at 2.5months.<br>Muscle biopsy at day 10 – vacuoles.<br>Laryngeal endoscopy showed normal morphology but spasms occurred upon minimal stimulation of the laryngeal inlet.<br>EMG: myotonia.<br>No sodium channel blockers given. Diagnosis posthumous.			
6	Episodic stridor often with cyanosis noted from birth. Parents reported "ptosis" and later spasms of hands. Myotonia recognized. EMG myotonia confirmed. Laryngoscopy normal. Exam "hypertonia" but otherwise NAD. Normal development. No details on treatment or progress.	F	SCN4A<br>G1306E<br>Inheritance unknown	(4)
7	APGAR 10. Shortly after birth, episodic apneas and admitted ICU. Diagnosed laryngomalacia. Discharged with medical treatment of GORD. Frequent episodes of stridor and apnea persisted, associated with cyanosis. Laryngomalacia surgery but no improvement. 14 months, hospitalized during winter because of severe episode of cyanosis and apnea provoked by exposure to cold.<br>O/E muscle hypertrophy especially of sternocleidomastoids. Athletic appearance, limb movements limited by hypertrophy.<br>EMG myotonia. Muscle biopsy: enlarged fibers.<br>Mexiletine started with some improvement in stiffness.<br>Between 2-8 years episodes of diplopia, strabismus, and dysphagia for cold drinks.<br>Age 8 episodes of sweating and tachycardia induced by cold.<br>Later more typical myotonia, legs hours to days, some warm up. Muscle hypertrophy.	M	SCN4A<br>G1306E<br>De novo	(5)
8	Oligohydramnios and intrauterine growth retardation. APGAR score 10. Parents noted breathing problems from birth. Daily apneas. Hospital at one month. Diagnosed GORD. Daily dyspnea persisted. ICU 3months – noted apneic episodes were assoc. with initial stridor followed by generalized stiffness, facial contraction, cyanosis, and bradycardia, generally without LOC. Episodes lasted a few seconds, with rapid recovery.<br>O/E peripheral hypertonia. Muscle hypertrophy. Clenched hands.<br>Rx CBZ with reduced episodes of dyspnea.	M	SCN4A<br>G1306E<br>De novo	(5)
9	Three generation family of PMC. Normal birth and APGAR score. Stridor and feeding problems within 24hrs. NG tube. Intermittent oxygen for desaturations when attempting to feed or cry. Diagnosed: laryngomalacia. Continuos stridor until 6months. Mild delay in motor milestones. Typical symptoms of PMC from 23 months. Intermittent stridor e.g. If crying, viral illness still observed at age 4.	M	SCN4A<br>T1313M<br>AD	(6)
10	Five generation family of SCM. 6yr old boy with episodes of hypoventilation, some with cyanosis from birth. Disappeared by age 1month. Difficulty drinking milk in first year. Difficulty running at age two. More typical myotonia from age 5 with muscle hypertrophy Great Aunt reportedly had similar cyanotic attacks but died age 1 from pneumonia.	1 M<br>1 F	SCN4A<br>Q1663E<br>AD	(7)
11	Spontaneous vaginal delivery 32/40. ICU care for four weeks. Presented at 8weeks with acute life threatening events (ALTE)s. Triggered by discomfort or crying, generalized stiffness with stridor, respiratory distress and ultimately apnea and bradycardia. 4 admissions to ICU for ALTEs. Muscle hypertrophy noted at 4months. EMG myotonia. Carbamazepine commenced. Instant cessation of ALTEs. Eye closure myotonia present age 2.5 years.	F	SCN4A<br>G1306E<br>De novo	(8)
12	Full term birth. Oxygen therapy required but no resuscitation. Presented at 6 weeks with recurrent laryngospasm associated with general	F	SCN4A	(8)

	stiffness, cyanosis and occasionally LOC. Laryngoscopy normal. Mild learning difficulties. Muscle hypertrophy noted at 10months. Recurrent laryngospasms until age 6 when carbamazepine given. Complete cessation of laryngospasms and improved stiffness. Genetic diagnosis confirmed age 15.		G1306E Presumed de novo	
13	Normal early history. Presented at 10months stridor and feeding difficulties. Choking with feeds recorded until age 3. Muscle hypertrophy noted age 6 with grip myotonia. Carbamazepine started with good effect on myotonia.	F	SCN4A G1306E AD	(8)
14	Normal birth history. Presented at 7days with episodes of apnea and stiffness. Duration of episodes increased at 11months. Spontaneously resolved but at age 2 demonstrated more typical myotonia exacerbated by cold. Age 5 muscle hypertrophy and some dysmorphic features including low set ears, long philtrum and puckered lips. Some improvement in stiffness with mexiletine, acetazolamide and phenytoin.	M	SCN4A I693L De novo	(9)
15	At birth: hypotonia with swallowing difficulties and respiratory distress requiring oxygen. Presented at 6months with recurrent apneas and general stiffness often induced by crying. Multiple hospital admissions for apnoea. Dysmorphic features high forehead, down-slanting palpebral fissures, low-set ears, high-arched palate, short neck, and barrel chest. EMG florid myotonia. With increasing age apnoeas less frequent but general myotonia more troublesome. Limited response to carbamazepine, mexiletine and acetazolamide. Good response to flecainide.	F	SCN4A G1306E De novo	(10)

Supplemental Table: Summary of published reports of infants with gain of function *SCN4A* mutations who did not die of SIDS but did experience severe respiratory compromise due to the effect of the mutation on their respiratory muscles.

Reference List

(1) Gay S, Dupuis D, Faivre L, et al. Severe neonatal non-dystrophic myotonia secondary to a novel mutation of the voltage-gated sodium channel (SCN4A) gene. *Am J Med Genet A* 2008 Feb 1;146:380-383.

(2) Furby A, Vicart S, Camdessanche JP, et al. Heterozygous CLCN1 mutations can modulate phenotype in sodium channel myotonia. *Neuromuscul Disord* 2014 Nov;24:953-959.

(3) Lion-Francois L, Mignot C, Vicart S, et al. Severe neonatal episodic laryngospasm due to de novo SCN4A mutations: a new treatable disorder. *Neurology* 2010 Aug 17;75:641-645.

(4) Brandt-Wouters E, Klinkenberg S, Roelfsema V, Ginjaar IB, Faber CG, Nicolai J. Teaching Video NeuroImages: sodium channel myotonia can present with stridor. *Neurology* 2013 Mar 5;80:e108.

(5) Caietta E, Milh M, Sternberg D, et al. Diagnosis and outcome of SCN4A-related severe neonatal episodic laryngospasm (SNEL): 2 new cases. *Pediatrics* 2013 Sep;132:e784-e787.

(6) Matthews E, Manzur AY, Sud R, Muntoni F, Hanna MG. Stridor as a neonatal presentation of skeletal muscle sodium channelopathy. *Arch Neurol* 2011 Jan;68:127-129.

(7) Kubota T, Kinoshita M, Sasaki R, et al. New mutation of the Na channel in the severe form of potassium-aggravated myotonia. *Muscle Nerve* 2009 May;39:666-673.

(8) Singh RR, Tan SV, Hanna MG, Robb SA, Clarke A, Jungbluth H. Mutations in SCN4A: a rare but treatable cause of recurrent life-threatening laryngospasm. *Pediatrics* 2014 Nov;134:e1447-e1450.

(9) Yoshinaga H, Sakoda S, Good JM, et al. A novel mutation in SCN4A causes severe myotonia and school-age-onset paralytic episodes. *J Neurol Sci* 2012 Apr 15;315:15-19.

(10) Portaro S, Rodolico C, Sinicropi S, Musumeci O, Valenzise M, Toscano A. Flecainide-Responsive Myotonia Permanens With SNEL Onset: A New Case and Literature Review. *Pediatrics* 2016 Apr;137.

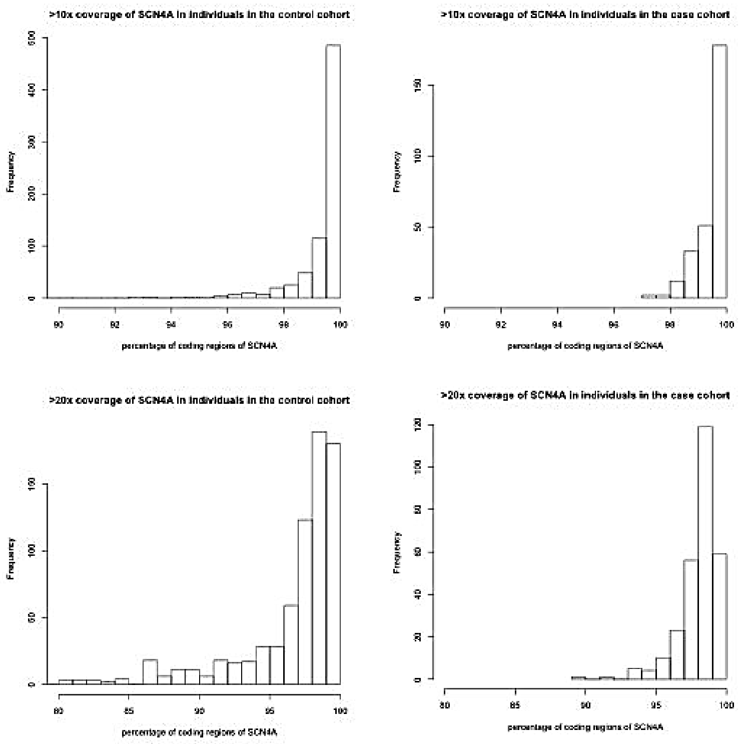

Supplemental Figure: Coverge of SCN4A coding regions in cases and controls

Clone	Activation					Fast Inactivation				
	N	I_{Peak} @0 mV (pA/pF)	N	$V_{1/2}$ (mV)	V_{slope} (mV)	$V_{1/2}$ (mV)	V_{slope} (mV)	Tau (@0mV) (ms)	N	$T_{Recovery}$ (ms)
**WT**	149	-127.5±6.4	146	-19.5±0.2	6.4±0.1	-65.3±0.3	5.4±0.0	0.30±0.00	105	5.63±0.14
**SIDS cohort**										
**S682W**	19	-94.2±9.9	17	-21.2±0.7	7.2±0.2	-67.0±0.6	5.9±0.2	0.40±0.02	17	6.15±0.33
**p**		0.124294		1	0.000376	0.016772	0.001384	2.22E-07		0.168144
**G859R**	18	-138.5±16.4	17	-20.2±0.8	5.7±0.2	-64.2±0.6	5.1±0.1	0.31±0.01	17	5.20±0.30
**p**		0.400129		1	0.013156	0.184117	0.015614	0.238201		0.218743
**V1442M**	14	-145.5±21.7	14	-21.7±0.8	6.3±0.2	-71.9±1.0	5.2±0.1	0.27±0.01	13	8.51±0.46
**p**		0.419262		0.297413	0.617672	2.65E-08	0.172465	0.069545		0.000007
**R1463S**	27	-71.3±8.3	25	-17.4±0.5	6.7±0.2	-61.7±0.6	6.3±0.1	0.31±0.01	19	1.95±0.09
**p**		0.000054		1	0.176747	2.23E-05	2.86E-08	0.077077		1.98E-11
**M1493V**	17	-78.2±8.3	17	-19.2±0.5	6.7±0.3	-65.3±0.8	5.5±0.2	0.29±0.01	7	5.76±0.47
**p**		0.00681		1	0.727018	0.928799	0.456311	0.997281		0.611244
**E1520K**	39	-49.8±9.7	28	-20.3±0.4	6.3±0.1	-66.3±0.5	5.4±0.2	0.33±0.01	20	5.63±0.29
**p**		2.1692E-14		1	0.305351	0.052083	0.880326	0.002981		0.9519948
**Control cohort**										
**R179Q**	11	-117.6±11.0	11	-19.3±0.6	6.5±0.2	-65.1±0.7	5.1±0.1	0.25±0.01	11	4.73±0.30
**p**		0.796829		1	0.639217	0.950686	0.063294	0.001666		0.063414
**R190W**	14	-153.4±21.8	14	-20.7±0.5	6.4±0.2	-64.9±0.5	5.3±0.1	0.29±0.01	13	5.09±0.20
**p**		0.188324		0.040354	0.79027	0.877528	0.993083	0.621112		0.243518
**L227F**	12	-107.7±16.1	12	-17.6±0.4	6.5±0.2	-67.8±0.8	5.6±0.1	0.33±0.02	10	5.33±0.30
**p**		0.542477		1	0.826592	0.0053782	0.186312	0.043428		0.604315
**D334N**	10	-141.0±26.6	10	-21.0±0.9	6.4±0.2	-66.4±0.8	5.3±0.1	0.27±0.01	9	5.07±0.30
**p**		0.650085		1	0.854767	0.179324	0.885881	0.059176		0.327061
**G863R**	12	-124.6±15.8	12	-20.3±0.8	6.3±0.1	-66.1±0.7	5.2±0.2	0.29±0.01	10	5.71±0.21
**p**		0.649001		1	0.43464	0.257994	0.409821	0.800613		0.634426
**A870T**	16	-132.6±20.0	15	-20.0±0.6	6.4±0.1	-65.7±0.6	5.0±0.1	0.29±0.01	14	6.29±0.55
**p**		0.832093		1	0.833794	0.509397	0.009465	0.975819		0.2172056
**M897K**	10	-102.8±23.3	9	-20.4±0.7	6.8±0.3	-67.7±1.3	5.5±0.2	0.31±0.02	7	6.21±0.54
**p**		0.263127		1	0.311082	0.065002	0.485371	0.911255		0.285506
**V1590I**	8	-92.8±13.2	8	-20.7±0.6	6.8±0.2	-65.8±0.9	5.4±0.2	0.30±0.02	7	5.73±0.61
**p**		0.288124		1	0.199203	0.416971	0.702681	0.961794		0.683139

Supplemental table 2. The exact p values of the data.

Shapiro-Wilk's normality test of residuals found all parameters except $V_{1/2}$ of activation to be non-normally distributed. Heteroscedasticity of variance was tested using Levene's test. Un-equal variance was found in all groups except $V_{1/2}$ for inactivation. For parameters with non-normally distributed data the Kruskal-Wallis rank sum test was performed with Dunn's pairwise multiple comparisons between the mean of each variant against the wild-type mean . For $V_{1/2}$ of activation a one-way ANOVA was performed with Games-Howell's post hoc test (un-equal variance) to compare the mean of each variant against the wild-type mean. Bonferroni correction was used to correct for multiple comparisons across all parameters (98 tests in total (14 tests*7 parameters). Bonferroni threshold across all parameters is $p=0.00051$.

Gene	NCBI mRNA Ref_Seq	Associated With SIDS
Major Channelopathy Genes		
KCNQ1	NM_000218	yes
KCNH2	NM_000238	yes
SCN5A	NM_198056	yes
RYR2	NM_001035.2	yes
Minor Channelopathy Genes		
AKAP9	NM_005751	
ANK2	NM_001148	
CACNA1C	NM_000719	
CACNA2D1	NM_000722	
CACNB2	NM_201590	
CALM1	NM_006888	
CALM2	NM_001743	
CALM3	NM_005184	
CASQ2	NM_001232.3	
CAV3	NM_001234	yes
DPP6	NM_130797	
GJA1	NM_000165	yes
GPD1L	NM_015141	yes
HCN4	NM_005477	
KCND3	NM_004980	yes
KCNE1	NM_001270402	
KCNE2	NM_172201	
KCNE3	NM_005472	
KCNJ2	NM_000891	
KCNJ5	NM_000890	
KCNJ8	NM_004982	yes
RANGRF	NM_016492	
SCN1B	NM_001037	
SCN3B	NM_018400	yes
SCN4B	NM_001142349	yes
SNTA1	NM_003098	yes
TRDN	NM_001256021	
Cardiomyopathy Genes		
AARS2	NM_020745	
ABCC9	NM_005691	
ACTC1	NM_005159	
ACTN2	NM_001103	
ANKRD1	NM_014391	
BAG3	NM_004281	
CALR3	NM_145046	
CRYAB	NM_001885	
CSRP3	NM_003476	
CTNNA3	NM_013266	
DES	NM_001927	
DSC2	NM_024422	
DSG2	NM_001943	
DSP	NM_004415	
DTNA	NM_001390	
EYA4	NM_004100	

Gene	NCBI mRNA Ref_Seq	Associated With SIDS
FHL2	NM_001450	
FKTN	NM_006731	
GATAD1	NM_021167	
JPH2	NM_020433	
JUP	NM_002230	
LAMA4	NM_001105206	
LDB3	NM_007078	
LMNA	NM_170707	
MIB1	NM_020774	
MTO1	NM_133645	
MYBPC3	NM_000256	
MYH6	NM_002471	
MYH7	NM_000257	
MYL2	NM_000432	
MYL3	NM_000258	
MYLK2	NM_033118	
MYOM1	NM_003803	
MYOZ2	NM_016599	
MYPN	NM_032578	
NEBL	NM_006393	
NEXN	NM_144573	
PDLIM3	NM_001114107	
PKP2	NM_004572	
PLN	NM_002667	
PRDM16	NM_022114	
PRKAG2	NM_016203	
PSEN1	NM_000021	
PSEN2	NM_000447	
RBM20	NM_001134363	
SDHA	NM_004168	
SGCD	NM_001128209	
TAZ	NM_000116	
TCAP	NM_003673	
TGFB3	NM_003239	
TMEM43	NM_024334	
TMPO	NM_003276	
TNNC1	NM_003280	
TNNI3	NM_000363	
TNNT2	NM_000364	
TPM1	NM_000366	
TTN	NM_003319	
TXNRD2	NM_006440	
VCL	NM_014000	

Supplementary Table 3: List of 90 genes associated with inherited cardiac conditions including those shown to be associated with SIDS
